# A Matter of Choice: Inhibition of c-Rel Shifts Neuronal to Oligodendroglial Fate in Human Stem Cells

**DOI:** 10.3390/cells9041037

**Published:** 2020-04-22

**Authors:** Lucia Mercedes Ruiz-Perera, Johannes Friedrich Wilhelm Greiner, Christian Kaltschmidt, Barbara Kaltschmidt

**Affiliations:** 1 Molecular Neurobiology, University of Bielefeld, 33615 Bielefeld, Germany; lucia.ruiz@uni-bielefeld.de; 2 Department of Cell Biology, University of Bielefeld, 33615 Bielefeld, Germany; johannes.greiner@uni-bielefeld.de (J.F.W.G.); c.kaltschmidt@uni-bielefeld.de (C.K.)

**Keywords:** NF-κB-c-REL, oligodendroglial fate shift, pentoxifylline, MBP, survival

## Abstract

The molecular mechanisms underlying fate decisions of human neural stem cells (hNSCs) between neurogenesis and gliogenesis are critical during neuronal development and neurodegenerative diseases. Despite its crucial role in the murine nervous system, the potential role of the transcription factor NF-κB in the neuronal development of hNSCs is poorly understood. Here, we analyzed NF-κB subunit distribution during glutamatergic differentiation of hNSCs originating from neural crest-derived stem cells. We observed several peaks of specific NF-κB subunits. The most prominent nuclear peak was shown by c-REL subunit during a period of 2–5 days after differentiation onset. Furthermore, c-REL inhibition with pentoxifylline (PTXF) resulted in a complete shift towards oligodendroglial fate, as demonstrated by the presence of OLIG2^+^/O4^+^-oligodendrocytes, which showed *PDGFRα*, *NG2* and *MBP* at the transcript level. In addition c-REL impairment further produced a significant decrease in neuronal survival. Transplantation of PTXF-treated predifferentiated hNSCs into an ex vivo oxidative-stress-mediated demyelination model of mouse organotypic cerebellar slices further led to integration in the white matter and differentiation into MBP^+^ oligodendrocytes, validating their functionality and therapeutic potential. In summary, we present a human cellular model of neuronal differentiation exhibiting a novel essential function of NF-κB-c-REL in fate choice between neurogenesis and oligodendrogenesis which will potentially be relevant for multiple sclerosis and schizophrenia.

## 1. Introduction

The development, function and regeneration of the human brain crucially depend on the generation of neurons, astrocytes and oligodendrocytes by neural stem cells (NSCs) [1]. Differentiation of NSCs results in the equal proportion of neurons and glia observed in the human brain [2], with particular proportions between distinct glial cell populations. For instance, in the cerebral cortex, astrocytes correspond to 20%, microglia to 5% and oligodendrocytes to even 75% of all glial cells [3]. The mechanisms underlying brain development and composition during adulthood crucially depend on fate decisions of NSCs, which are also directly linked to the progression of neurodegenerative diseases. For instance, Alzheimer’s disease was recently correlated with the inability of NSCs to undergo neuronal differentiation [4]. In terms of multiple sclerosis, it has been suggested that the differentiation of NSCs into oligodendrocytes contributes to myelin repair, in addition to the role of resident oligodendrocyte precursor cells [5]. Moreover, cognitive ageing decline is correlated with a decrease in oligodendrocytes and neurons but not astrocytes [3], further indicating the importance of understanding the molecular mechanisms of lineage choice and their role in aging and disease.

The transcription factor nuclear factor kappa-light-chain-enhancer of activated B-cells (NF-κB) is involved in diverse cellular processes like inflammation or cell death, but also plays a critical role in the mammalian brain during development and adulthood. Especially the NF-κB subunit p65 was observed to have a predominant activity in the adult mouse brain, while its activity was directly related to synaptic function, transmission and plasticity [6,7,8,9], as well as axonal outgrowth and polarization [9,10]. NF-κB-p65 was also activated during neural differentiation of mouse NSCs, while its inhibition promoted self-renewal [11]. However, little is known about the role of NF-κB in the regulation of human NSCs during neuronal differentiation and their fate choice between neurogenesis and gliogenesis.

In the present study, we took advantage of a human cellular model, which we recently used to investigate the NF-κB p65-dependent neuroprotection in human neurons [12]. We isolated neural crest-derived stem cells (NCSCs) from the adult human nasal cavity [13,14], and differentiated them into NSCs and further into functional mature glutamatergic neurons according to our previous studies [12,15]. A comparable mouse model system was previously used to study fate shifts of NSCs originating from peripheral NCSCs [16]. These cells were reprogrammed in culture and obtained central nervous system characteristics, being further able to differentiate into myelinating oligodendrocytes. Applying this ideal model to study human neuronal development and maturation, we were able to determine that NF-κB-c-REL is significantly increased in early glutamatergic differentiation of human NCSC-derived NSCs, indicating that it might be involved in neuronal differentiation. Pentoxifylline (PTXF), an approved FDA drug which has been shown to deviate the immune response in multiple sclerosis patients [17], exerts its pharmacological effects through multiple targets in vivo. PTXF is a known inhibitor of c-REL nuclear translocation [18] that was shown to specifically reduce c-REL levels in T regulatory cells without affecting the other NF-κB subunits [19]. It also inhibits intracellular phosphodiesterases, leading to an increase in intracellular cAMP [20]. Thus, PTXF seems to be one of the best characterized pharmacological agents for c-REL inhibition (see patent W02017058881A1, 2016). Inhibition of c-REL by PTXF induced a direct shift from the neuronal towards the oligodendrocyte fate, as evidenced by the increase of the oligodendrocyte specific markers, oligodendrocyte transcription factor 2 (OLIG2), O4 and myelin basic protein (MBP), as well as the reduction in the expression of neuronal markers such as neurofilament 200 (NF200) and vesicular glutamate transporter 2 (VGLUT2), and the decrease in the expression of other glial markers like for glial fibrillary acidic protein (GFAP) and the stemness marker nestin. The nerve growth factor receptor p75 is a receptor for the neurotrophin NGF. p75 was upregulated during differentiation into the oligodendroglial fate. p75 was initially described as coreceptor of the trkA receptor, but it could also function as an autonomous signaling molecule activating NF-κB in a NGF-dependent manner [21,22].

Transplantation of PTXF-treated predifferentiated NSCs into mouse organotypic cerebellar slices further demonstrated their ability to differentiate into MBP^+^ oligodendrocytes and produce myelin ex vivo under appropriate environmental conditions. These findings strongly suggest that c-REL is necessary during the neuronal glutamatergic differentiation of hNSCs, being particularly important for neuronal survival; however, for the differentiation into the oligodendroglial fate, c-REL might be a repressor.

## 2. Materials and Methods

### 2.1. Isolation and Cultivation of Human NCSCs

NCSCs were isolated from adult human inferior turbinate tissue obtained by biopsy during routine surgery after informed consent according to local and international guidelines, and cultivated as described previously [13,14]. All experimental procedures were ethically approved by the ethics board of the medical faculty of the University of Münster (No. 2012–015-f-S).

### 2.2. Neuronal Differentiation of Human NCSCs to Glutamatergic Neurons

**Figure 1:**
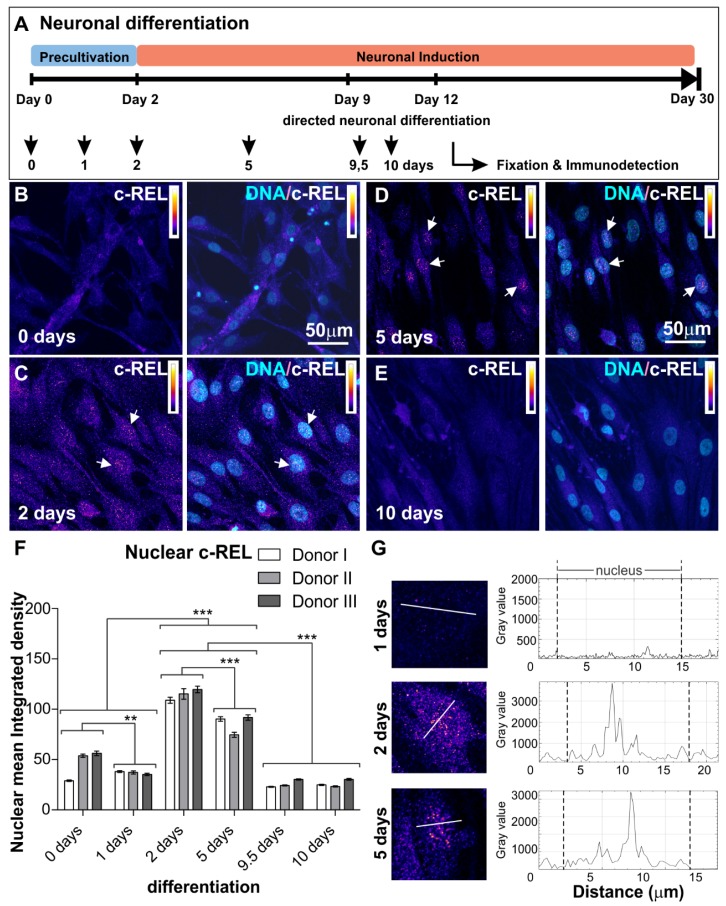
Immunocytochemical analysis of c-REL. (**A**) Neuronal differentiation procedure. Adult human NCSCs were differentiated into NSCs and further into glutamatergic neurons. NF-κB subunit composition was analyzed at different time points as indicated. (**B**–**E**) NCSC-derived NSCs labeled against c-REL after 0, 2, 5 and 10 days of glutamatergic differentiation respectively. Each panel shows c-REL (left) and DNA colocalization (right). Intensity scale indicating white and black as highest and lowest intensity levels. Arrows depict c-REL-nuclear activation. (**F**) Quantification of immunocytochemical analyses showing c-REL nuclear mean integrated density during early differentiation (*n* = 3, mean ± SEM). Normality was refuted using Shapiro-Wilk normality test. Nonparametric Kruskal-Wallis (*** *p* ≤ 0.001) and Bonferroni corrected post-test (*** *p* < 0.001) revealed significantly increased nuclear translocation of NF-κB-c-REL on days 2 and 5. (**G**) Fluorescence intensity profiles measured at three different time points (1, 2 and 5 days of differentiation) for cells following transects as shown clearly revealed the difference between nuclear and cytoplasmic fluorescence. NCSCs: neural crest-derived stem cells, NSCs: neural stem cells.

For the guided differentiation of NCSCs to NSCs and further to glutamatergic neurons, NCSCs cultivated as described above were exposed to neuronal induction medium as previously described [12,15]. For the detailed procedure, see Supplementary Material and Figure 1A.

### 2.3. Pentoxifylline Treatment

Pentoxifylline (PTXF) is a xanthine derivative and a potent inhibitor of NF-κB-c-REL, showing a specific effect on the c-REL subunit and not on other NF-κB subunits like p65 [18,19]. Thus, inhibition of c-REL-activity via PTXF-treatment was performed by adding 500 µg/mL PTXF to the neuronal differentiation media, after we determined that this concentration was suitable for our model [19]. PTXF was refreshed every 1–2 days for 30 days, while differentiating NSCs not exposed to PTXF were used as a control.

### 2.4. Cerebellar Slice Culture, Demyelination and Cell Transplantation

Organotypic cerebellar slice culture was based on published protocols [23,24,25]. Mice were decapitated and whole brain was removed and kept in ice cold Hanks’ buffered salt solution (HBSS). The cerebellum was dissected from mice at P10 under a dissecting microscope. Then, 400 µm Parasagittal Cerebellar slices were cut using a McIlwain tissue chopper, separated into individual slices and placed 4 per insert on collagen-coated cell culture inserts (Millicell, Merck Millipore, Burlington, MA, USA) in medium. Slices were cultured in serum-based medium containing 50% Opti-MEM, 25% HBSS, 25% heat-inactivated horse serum and supplemented with 2 mM Glutamax, 28 mM d-glucose, 100 U/mL penicillin/streptomycin and 25 mM HEPES, and cultivated at 37 °C and 5% CO_2_ in a humidified incubator. After 3 days in vitro (DIV), slices were transferred to serum-free medium consisting of 98% Neurobasal-A and 2% B-27 (Thermofisher Scientific, Waltham, MA, USA), supplemented with 2 mM Glutamax, 28 mM d-glucose, 100 U/mL penicillin/streptomycin and 25 mM HEPES. Half of the culture medium was exchanged with fresh medium every other day.

Demyelination was induced by oxidative stress at 14 DIV. For this, slice cultures were transferred to fresh serum-free medium containing 0.5 µM H_2_O_2_ (Sigma-Aldrich, Saint Louis, MO, USA) and incubated overnight for 18 h [26]. After incubation, slices were either washed with PBS and fixated with PFA4% for immunocytochemistry, or further transferred to serum-free medium without H_2_O_2_ for further cultivation and cell transplantation. Undifferentiated or predifferentiated NCSC-derived NSCs (treated with PTXF for 3 days, +PTXF) were transplanted into the slices (1 × 10^4^ cells were transplanted per slice) to determine their ability to differentiate into oligodendrocytes and to produce myelin in this demyelination model. After transplantation, slices were further cultivated for two weeks, fixated and stained using indirect immunodetection, as described below. Transplanted human cells were identified with antihuman nuclei (huNu) within the slices, and antimyelin basic protein (MBP) was used to visualize myelination by human oligodendrocytes.

### 2.5. Immunocytochemistry

Differentiated NCSCs were fixed in phosphate-buffered 4% paraformaldehyde (pH 7.4) for 15 min at room temperature (RT) following the immunocytochemical staining procedure described in [12]. For the detailed procedure, see Supplementary Material. The primary antibodies used against NF-κB subunits were anti-NF-kappa B p65 (1:100, sc-8008, Santa Cruz Biotechnology, Dallas, TX, USA; 1:200, D14E12, Cell Signaling, Danvers, MA, USA), anti-c-REL (1:100, sc-70x, Santa Cruz Biotechnology; 1:400, #4727, Cell Signaling), anti-RELB (1:100, sc-226, Santa Cruz Biotechnology; 1:1600, #10544, Cell Signaling), anti-p50 (1:100, sc-8414, Santa Cruz Biotechnology), anti-p52 (1:100, sc-298, Santa Cruz Biotechnology), anti-IκBα (1:100, sc-371, Santa Cruz Biotechnology); antibodies used as differentiation markers, antinestin (1:200, MAB5326, Millipore), antineurofilament 200 (NF200, 1:200, N4142, Sigma-Aldrich), anti-VGLUT2 (vesicular glutamate transporter 2, 1:200, MAB5504, Millipore), anti-OLIG2 (oligodendrocyte transcription factor 2, 1:250, Q13516, R&D Systems, Minneapolis, MN, USA), anti-O4 (1:100, IgM, R&D), Anti-αSMA (alpha smooth muscle actin,1:200, A5691, Sigma), anti-NGFRp75 (nerve growth factor receptor p75, 1:100, sc-6188, Santa Cruz), anti-GFAP (glial fibrillary acidic protein, 1:500, Z0334, DAKO, Santa Clara, CA, USA) and indicative of apoptosis, anticleaved caspase-3 (1:300, #9664, Cell Signaling). The secondary fluorochrome-conjugated antibodies were incubated for 1 h at RT. The secondary antibodies used were goat antimouse Alexa 555, goat antirabbit Alexa 555, donkey antigoat Alexa 555, and goat antimouse-IgM Alexa 555 (1:300, Life Technologies, Carlsbad, CA, USA). Nuclear counterstaining was performed with 49,6-diamidino-2-phenylindole (DAPI; 1 µg/mL; Sigma-Aldrich). Fluorescence imaging was performed using confocal laser scanning microscopy (LSM 780; Carl Zeiss, Jena, Germany) and analyzed using ZEN software (Carl Zeiss) or ImageJ (Bethesda, MD, USA).

Wholemount immunostaining of cerebellar organotypic slices was slightly modified from O’Sullivan et al., 2017. Slices were washed twice in PBS followed by fixation in 4% formaldehyde solution for 1 h at RT. Slices were washed twice in PBS and incubated overnight in a blocking buffer that consisted of 10% BSA and 0.5% Triton-X 100 in PBS. Slices were washed three times with 0.01% Triton-X100 in PBS and incubated for 24–48 h at 4 °C in primary antibody. Slices containing transplanted cells were double immuno-labeled with antihuman nuclei (huNu, 1:200, MAB1281, Millipore) to detect the transplanted human cells in mouse cerebellar slices, and combined with antimyelin basic protein (MBP, 1:200, rat monoclonal, Biorad) to analyze the white matter. For all antibody dilutions, PBS supplemented with 2% BSA and 0.1% Triton-X 100 was used. Slices were then washed three times with 0.01% Triton-X 100 in PBS and incubated for 24–48 h at 4 °C with secondary antibodies (goat antimouse Alexa 555, goat antirat Alexa 555, 1:300, Life Technologies). Then, slices were washed with PBS and incubated with DAPI (1:1000) for nuclear counterstaining, 1h at 4 °C in agitation. Slices were washed three times and mounted on glass cover slides in Mowiol-4-88. Fluorescence imaging was performed using a confocal laser scanning microscopy (LSM 780; Carl Zeiss).

### 2.6. Quantitative Polymerase Chain Reaction (qPCR)

Total RNA was isolated using NucleoSpin RNA kit (Macherey-Nagel, Düren, Germany) according to the manufacturer’s guidelines. The quality and concentration of RNA were assessed via Nanodrop ultraviolet spectrophotometry followed by cDNA synthesis using First Strand cDNA Synthesis Kit (Fermentas, Thermofisher Scientific, Waltham, MA, USA). qPCR were performed in triplicate using PerfeCTa SYBR^®^ Green SuperMix (Quantabio, Beverly, MA, USA) according to the manufacturer’s guidelines, and assayed with a Rotor Gene 6000 (Qiagen, Venlo, Netherlands). Primers are listed in Supplementary Table S1.

### 2.7. Image Analyses and Quantification

Fluorescence imaging was performed using a confocal laser scanning microscopy (LSM 780; Carl Zeiss). Image acquisition settings were kept constant across treatments. For the analysis of nuclear NF-κB protein, quantification of indirect immunofluorescence was performed for a minimum of 3 different donors by analyzing 6–12 pictures per time-point and donor. Mean nuclear integrated density was measured by defining the region of interest with the nuclear counterstaining using ImageJ. To illustrate the pattern of all the NF-κB subunits together, we used a polynomial function fitting the individual data normalized by the range of the median. This was determined using custom-written MATLAB routines (R2016b 64 bit, MathWorks, Natick, MA, USA).

For the analysis of neuronal survival, the amount of nonviable neurons recognized by nuclear condensation and/or fragmented chromatin was counted and the death rate was calculated as previously described [12].

Three cerebellar slices were analyzed per condition for confocal imaging. The quantification of cells was performed in a similar area for all slices in the central region corresponding to cerebellar nuclei. The number of huNu^+^, huNu^+^MBP^+^ and total cells were counted per image, and the labeling index was calculated. The proportion of labeled cells was determined as the number of labeled cells (number of positive cells for the antibody or antibodies of interest) over the total number of cells observed × 100. The ratio of huNu^+^ and double positive huNu^+^MBP^+^ was averaged for each condition.

### 2.8. Statistical Analyses

A statistical analysis was performed using Past3 and GraphPad Prism 5 (GraphPad Software, La Jolla, CA, USA). Normality was refuted using Shapiro-Wilk normality test. Homogeneity of variance was tested using Levene’s test, and a nonparametric Kruskal-Wallis test was applied to compare medians between different donors (*** *p* value < 0.001). A nonparametric Mann-Whitney test was used to compare two pair of groups (*** *p* ≤ 0.001). A Tukey’s test or Bonferroni corrected post-test served to identify the significance of the differences between the groups, by comparing the medians or means respectively (* *p* ≤ 0.05, ** *p* ≤ 0.01, *** *p* ≤ 0.001).

## 3. Results

### 3.1. NF-κB-c-REL during Early Stages of Glutamatergic Differentiation in NCSC-Derived NSCs

**Figure 2:**
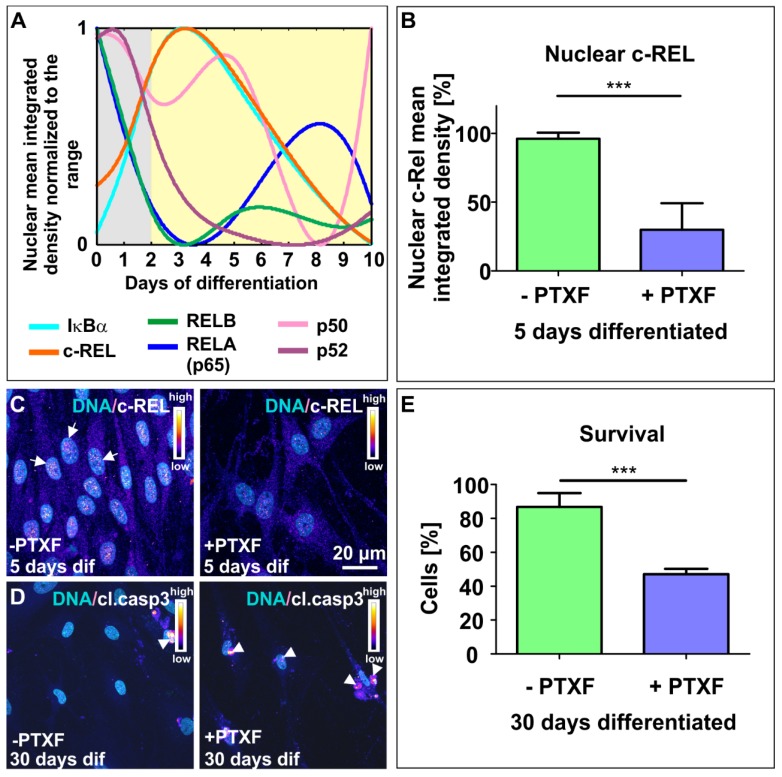
NF-κB subunit composition in early glutamatergic differentiation and c-REL potential function. (**A**) NF-κB subunit distribution in early stages of glutamatergic differentiation. Nuclear mean integrated density illustrated by fitting a polynomial function of the individual data normalized by the range of the median, showing each of the NF-κB subunits, as indicated by colors. (**B**) Quantified c-REL nuclear mean integrated density (*n* = 3, mean ± SEM). Nonparametric Mann Whitney test confirmed a significant decrease in nuclear c-REL after 5 days of differentiation with PTXF (*** *p* ≤ 0.0001), a known c-REL inhibitor. (**C**) 5 days differentiated NCSC-derived NSCs in the absence or presence of PTXF, labeled against c-REL shown by intensity scale and DNA colocalization. Arrows depict c-REL nuclear activation. (**D**) NCSC-derived NSCs differentiated in the absence or presence of PTXF for 30 days, labeled against cleaved-caspase-3 (cl.casp-3, intensity scale) showing DNA colocalization. Arrowheads depict dead cells. (**E**) Survival quantified for NCSC-derived NSCs differentiated in the absence or presence of PTXF for 30 days (*n* = 3, mean ± SEM). Nonparametric Mann Whitney test confirmed a significant decrease in survival PTXF-treated (*** *p* ≤ 0.001) compared to untreated neurons. PTXF: pentoxifylline, NCSCs: neural crest-derived stem cells, NSCs: neural stem cells.

NSCs originating from neural crest-derived stem cells were exposed to neuronal differentiation conditions for up to ten days according to our previously described protocol [12] (see Figure 1A for overview). The activities of NF-κB subunits p65, RELB, c-REL, p50 and p52 (Figure S1A) were characterized by assessing their nuclear localization (Figure S1B) by immunocytochemistry. We observed low levels of nuclear p65 protein, which even revealed a further significant decrease during early neuronal differentiation for up to ten days (Figure S2). A transient increase in nuclear RELB on day one of differentiation was accompanied by elevated protein levels of nuclear p52, which were found to be significantly reduced after 24 h and during further neuronal differentiation (Figures S3 and S4). While p50 protein levels showed no relevant differences during neuronal differentiation (Figure S5), levels of IκBα protein were elevated from day 2 to day 5 of neuronal differentiation, indicating inactivation of RELB/p52 dimers in an IκBα-dependent manner (Figure S6). Notably, a significant increase of nuclear c-REL protein was observable on day 2 of neuronal differentiation, when activation of c-REL started by translocation into the nucleus (Figure 1B–G). On day 5, nuclear c-REL was elevated, with a significant reduction in comparison to day 2 (Figure 1B–G) and a further decrease later on (Figure 1B–G). In summary, our findings indicate an opposing transient nuclear increase from RELB/p52 initially, towards a c-REL-increase during the early phase of neuronal differentiation of hNSCs (Figure 2A).

### 3.2. NF-κB-c-REL is Crucial for Cell Survival during Glutamatergic Differentiation of Adult Human Stem Cells

**Figure 3:**
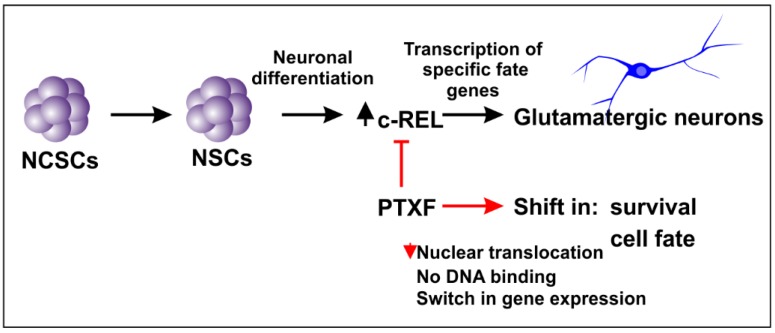
Diagram showing neuronal differentiation of NCSCs and PTXF mechanisms. The nuclear increase of NF-κB-c-REL directs differentiation into the glutamatergic neuronal fate. c-Rel inhibition by PTXF leads to a switch in the gene expression program from the neuronal fate, resulting in the cell fate shift, cell death or both. PTXF: pentoxifylline, NCSCs: neural crest-derived stem cells, NSCs: neural stem cells.

To further determine the role of NF-κB-c-REL during differentiation of NCSC-derived NSCs into glutamatergic neurons, we exposed differentiating NSCs to the c-REL-inhibitor and FDA-approved drug pentoxifylline (PTXF) [19]. In order to achieve adequate inhibition of c-REL activity, and to minimize the potentially undesirable indirect effects inherent to pharmacological treatment like the effected dose and cell-type specificity, we tested different concentrations of PTXF during the 5 days of differentiation in PTXF-treated differentiating NSCs. Thus, we confirmed that PTXF caused an important decrease of nuclear c-REL protein in PTXF-treated differentiating NSCs compared to untreated controls (96.11% ± 4.48%, −PTXF; 29.93% ± 3.71%, +PTXF, Figure 2B,C, nonparametric Mann Whitney test, *** *p* ≤ 0.0001), finding the ideal effect at 500 µg/mL of medium. Although a signal could still be detected, inhibition of c-REL-activity was evident (Figure 2B,C). Notably, PTXF-treatment resulted in increased amounts of cleaved-caspase 3 positive neurons after prolonged differentiation into glutamatergic neurons compared to control (Figure 2D). The quantification of neuronally differentiated cells showing nuclear condensation and/or fragmented chromatin confirmed a significant decrease in the survival of the PTXF-treated differentiated glutamatergic neurons (47.14% ± 3.17% survival, Figure 2E) compared to untreated neurons (86.83% ± 8.16% survival, Figure 2E, nonparametric Mann Whitney test, *** *p* ≤ 0.001). In addition, we evaluated apoptosis after 5 days of differentiation, and determined that the death rate on day 5 was similar in differentiating NSCs treated with PTXF (5.73% ± 1.27%, Figure S10), compared to untreated control cells (5.64% ± 1.54%, nonparametric Mann Whitney test, *p* = 0.8728, Figure S10), showing no significant differences and further indicating that treated cells begin to initiate the apoptotic program later. These findings demonstrate that NF-κB-c-REL plays a central role in cell survival during differentiation into the neuronal fate and raises the question of whether this may be accompanied by a shift in cell fate (Figure 3).

### 3.3. c-REL Inhibition by Pentoxifylline Induces a Shift into the Oligodendrocyte Fate

**Figure 4:**
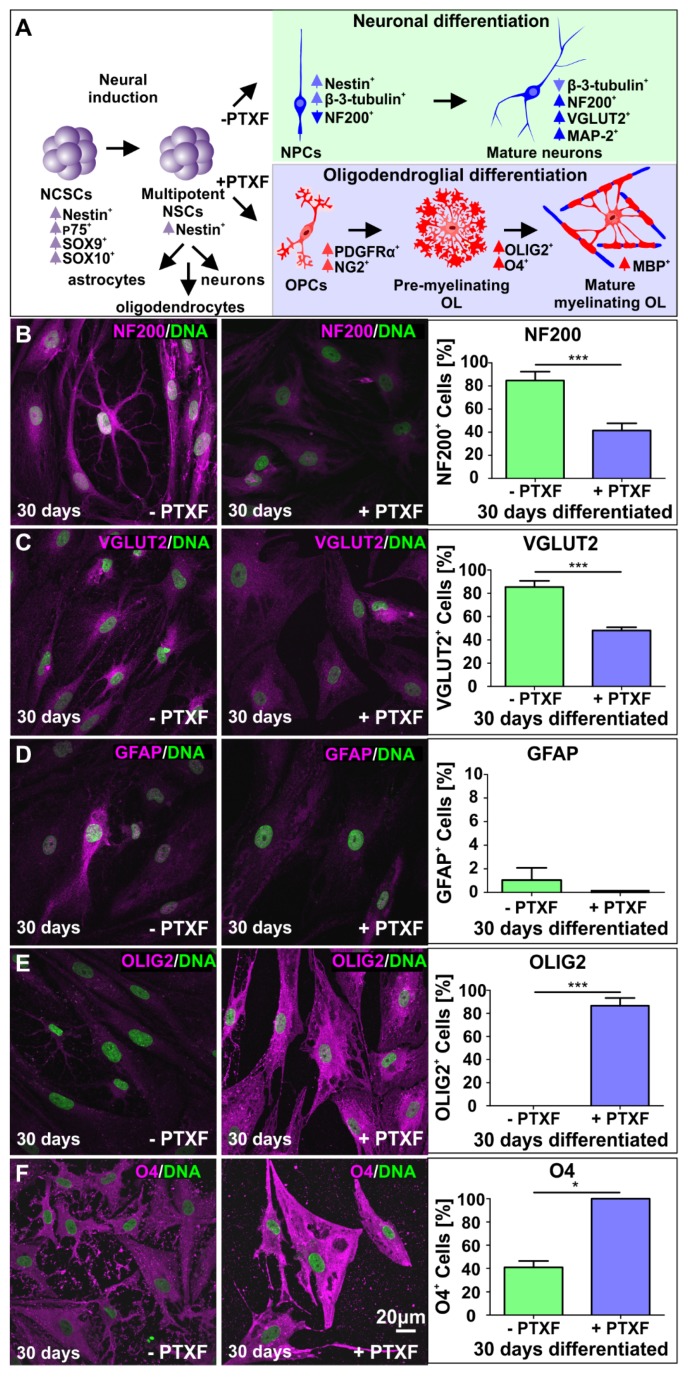
Immunocytochemical analysis of neuronal and glial markers after glutamatergic differentiation for 30 days in the presence or absence of pentoxifylline. (**A**) Transcription expression changes during specification of NCSCs into multipotent NSCs with the ability to differentiate into astrocytes, oligodendrocytes and neurons. Differentiation in the absence or presence of PTXF directed the cell fate into the neuronal (light green) or oligodendroglial fate (light blue), further analyzed by the expression of particular markers as indicated (modified from [5,13]). (**B**) Differentiated NCSC-derived NSCs labeled against NF200 and quantification showing the percentage of NF200^+^ neurons. Nonparametric Kruskal-Wallis test (*** *p* < 0.0005) revealed a significant decrease of NF200^+^ neurons in PTXF-treated cells (41.44% ± 6.31%) compared to untreated neurons (84.68% ± 7.70%). (**C**) Differentiated NCSC-derived NSCs labeled against VGLUT2, and quantification of VGLUT2^+^ neurons. Nonparametric Kruskal-Wallis test (*** *p* < 0.0005) confirmed a significant decrease of VGLUT2^+^ neurons in the differentiated PTXF-treated cells (47.97% ± 2.72%) compared to untreated neurons (85.37% ± 5.34%). (**D**) Differentiated NCSC-derived NSCs labeled against GFAP (astrocyte marker) and respective quantified percentage of GFAP^+^ cells, indicating a very low number of astrocytes present in the untreated control (1.04% ± 1.04%) which are completely absent in the differentiated PTXF-treated cells (0%, nonparametric Kruskal-Wallis test *p* = 0.3173, no significance). (**E**) Differentiated NCSC-derived NSCs labeled against OLIG2 (early oligodendrocyte marker) and quantified percentage of OLIG2^+^ cells, showing no positive cells in untreated control neurons (0%), while most differentiated PTXF-treated-NSCs OLIG2^+^ (86.67% ± 6.67%). Nonparametric Kruskal-Wallis test (*** *p* < 0.001) confirmed significant increase of OLIG2^+^ cells in PTXF-treated differentiated cells, indicating a shift into the oligodendrocyte fate. (**F**) Differentiated NCSC-derived NSCs labeled against O4 (immature oligodendrocyte marker) and quantification of O4^+^ cells, showing a significant increase of O4^+^ cells in PTXF-treated differentiated NSCs (100%) as shown by nonparametric Kruskal-Wallis test (* *p* < 0.05), compared to untreated neurons (40.97% ± 5.42%). PTXF: pentoxifylline, NCSCs: neural crest-derived stem cells, NSCs: neural stem cells, OPCs: Oligodendrocyte precursor cells, OL: oligodendrocytes, NF200: Neurofilament 200.

We investigated potential c-REL-dependent cell fate shifts in differentiating NSCs exposed to PTXF by analyzing mature neuronal and glial markers as indicated in Figure 4A. Here, treatment of differentiating NSCs with PTXF led to significantly reduced amounts of neurofilament 200 positive (NF200^+^) cells in comparison to control (Figure 4B; 41.44% ± 6.31% +PTXF and 84.68% ± 7.70% −PTXF, Kruskal-Wallis test *p* < 0.0005). Similar results were found for VGLUT2^+^ cells (Figure 4C; 47.97% ± 2.72% +PTXF and 85.37% ± 5.34% −PTXF, nonparametric Kruskal-Wallis test *p* < 0.0005).

**Figure 5:**
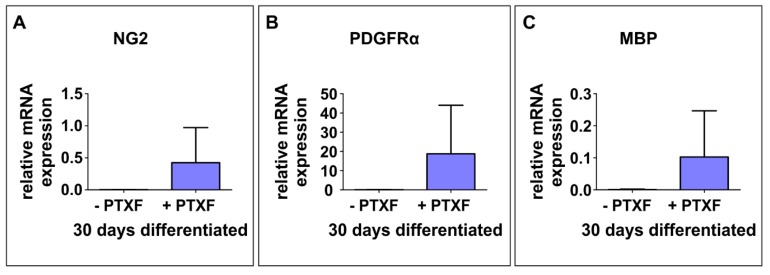
Early c-REL inhibition leads to a cell fate shift into the oligodendrocyte fate. (**A**–**C**) Real time polymerase chain reaction showing relative mRNA levels of Oligodendrocyte markers transmembrane proteoglycan nerve-glia antigen 2 (*NG2*), Platelet derived growth factor receptor alpha (*PDGFRα*), and Myelin basic protein (*MBP*) respectively, relative to the mean value of *GAPDH* and *RPLP0*. These early (*NG2*, *PDGFRα*) and mature oligodendrocyte transcripts (*MBP*) are only present in differentiated PTXF-treated NCSC-derived NSCs, indicating a shift into the oligodendrocyte phenotype (*n* = 2, mean ± SD). PTXF: pentoxifylline, NCSCs: neural crest-derived stem cells, NSCs: neural stem cells.

Interestingly, PTXF-treated differentiating NSCs were completely negative for glial fibrillary acidic protein (GFAP), suggesting the absence of radial glia and astrocytes (1.04% ± 1.04%, −PTXF; 0%, +PTXF, nonparametric Kruskal-Wallis test *p* = 0.3173, Figure 4D). In contrast, PTXF-treated NSCs underwent differentiation into the oligodendrocyte lineage, as demonstrated by a complete and highly significant shift towards OLIG2^+^ cells (86.67% ± 6.67%, +PTXF, nonparametric Kruskal-Wallis test, *** *p* < 0.001, Figure 4E) which were not detectable in the untreated control (0%, −PTXF, Figure 4E). Significantly increased amounts of O4^+^ cells after PTXF-treatment compared to untreated control (100%, +PTXF; 40.97% ± 5.42%, −PTXF, nonparametric Kruskal-Wallis test, *** *p* < 0.001, Figure 4F) were accompanied by the presence of Platelet derived growth factor receptor alpha (*PDGFRα*), transmembrane proteoglycan nerve-glia antigen 2 (*NG2*) and Myelin basic protein (*MBP*) at the mRNA level, which were totally absent in untreated neurons (Figure 5A–C). The observed cell fate shift was further confirmed by a major change in the cellular morphology of the PTXF-treated cells, which acquired a polygonal-like shape with an extended cell surface, as well as a reduction of cellular processes compared to control (Figure 4B–F). In addition, upon PTXF-treatment, we observed a slightly increased amount of Nestin^+^ cells (5.68% ± 1.33%, +PTXF; 2.48% ± 1.44%, −PTXF, nonparametric Kruskal-Wallis test, *p* = 0.2683, Figure S8A) and p75^+^ cells (46.67% ± 29.06%, +PTXF; 8.25% ± 6.03%,−PTXF, nonparametric Kruskal-Wallis test, *p* = 0.3758, Figure S8B). These results indicate that a small population of cells are Nestin^+^ positive, suggesting that these have a rather undifferentiated state compared to NSCs-derived glutamatergic neurons, whereas p75^+^ cells have a more differentiated state, directed into the oligodendrocyte lineage, as p75 is also a marker for adult human oligodendrocytes [22]. PTXF-treated and untreated NSCs were further found to be negative for alpha smooth muscle actin (αSMA), indicating the absence of mesodermal cells and a respective fate switch towards the mesodermal lineage (Figure S8C). In the absence of NF-κB-c-REL, a fate shift was observed from glutamatergic neurons towards oligodendrocytes.

### 3.4. Transplanted PTXF-Treated Predifferentiated NSCs into Demyelinated Mouse Organotypic Cerebellar Slices Integrate and Differentiate into Myelinating Oligodendrocytes

**Figure 6:**
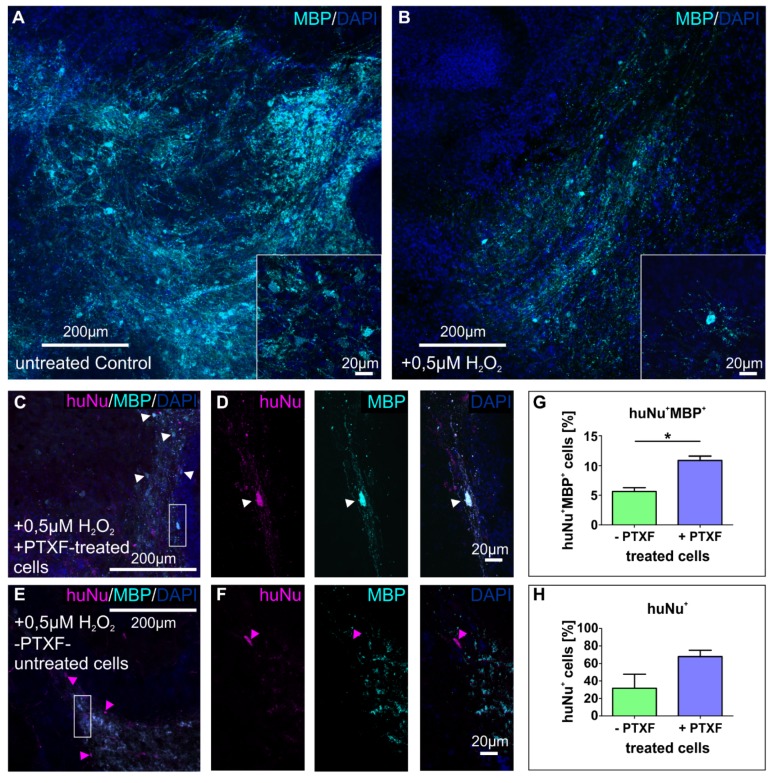
Immunocytochemical analyses of transplanted NCSC-derived NSCs or predifferentiated NCSC-derived NSCs into demyelinated mouse organotypic cerebellar slice cultures. (**A**,**B**) Immunostaining against MBP showing untreated control and 0.5 µM H_2_O_2_ treated cerebellar mouse slices. Confocal images show a clear decrease in MBP fluorescence at 0.5 µM H_2_O_2_ compared to control in a representative area. Insets show a central area of A and B respectively under higher magnification. Undifferentiated or predifferentiated NCSC-derived NSCs (treated with PTXF for 3 days, +PTXF) were transplanted into the slices and further cultivated for 2 weeks to determine their ability to differentiate and produce myelin in this demyelination model. (**C**) Immunostaining against MBP and huNu showing a 0.5 µM H_2_O_2_ treated cerebellar mouse slice transplanted with predifferentiated NCSC-derived NSCs (+PTXF-treated cells). White arrowheads indicate huNu^+^MBP^+^ double positive cells, representing cells of human origin which express MBP, human oligodendrocytes. Inset shows a clear huNu^+^MBP^+^ cell indicating its human origin. (**D**) huNu^+^MBP^+^ cell from the inset in C, at higher magnification showing this cell from human origin expressed MBP at the protein level. (**E**) Immunostaining against MBP and huNu showing a 0.5 µM H_2_O_2_ treated cerebellar mouse slice transplanted with untreated NCSC-derived NSCs (−PTXF-untreated cells). Magenta arrowheads indicate huNu^+^ cells of human origin. Inset shows a huNu positive cell indicating its human origin. (**F**) Inset indicated in E, at higher magnification showing a huNu^+^MBP^−^ NCSC-derived NSCs. (**G**) Quantification showing the percentage of huNu^+^MBP^+^ double positive cells in H_2_O_2_-treated cerebellar mouse slices transplanted with untreated NCSC-derived NSCs (−PTXF) or with predifferentiated NCSC-derived NSCs (+PTXF), showing the ratio of MBP^+^ cells of human origin present after 2 weeks of transplantation. Nonparametric Kruskal-Wallis test (* *p* < 0.05) shows a significant increase of MBP^+^ cells of human origin in slices transplanted with predifferentiated NSCs (10.87% ± 0.73%, +PTXF), compared to slices transplanted with untreated NSCs (5.63% ± 0.64%, −PTXF). (**H**) Quantification of huNu^+^ cells in H_2_O_2_-treated cerebellar mouse slices transplanted with untreated NCSC-derived NSCs (31.71% ± 16.02%, −PTXF) or with predifferentiated NCSC-derived NSCs (67.87% ± 7.23%, +PTXF), indicating the proportion of cells from human origin present after 2 weeks of transplantation. No significant differences were observed in the ratio of huNu^+^ cells according to nonparametric Kruskal-Wallis test *p* = 0.1266. NCSCs: neural crest-derived stem cells, NSCs: neural stem cells, MBP: myelin basic protein, H_2_O_2_: hydrogen peroxide, PTXF: pentoxifylline, huNu: human Nuclei.

In order to determine the capability of predifferentiated NSCs treated with PTXF for 3 days (+PTXF) to develop oligodendrocytes under a neural environment, we used a known ex vivo demyelination model induced by oxidative stress using H_2_O_2_ in cultured mouse organotypic cerebellar slices [26]. We first confirmed the oxidative stress-mediated demyelination by observing a clear decrease in myelin basic protein (MBP) fluorescence in the major white matter tract in H_2_O_2_-treated cerebellar mouse slices compared to untreated control slices (Figure 6A,B). Transplanted cells were identified using an antihuman nuclei antibody (huNu) to recognize human cells within the mouse cerebellar slices. Transplanted predifferentiated NSCs were able to integrate in the white matter, where they exhibited a clear oligodendroglial phenotype and differentiated into MBP^+^ oligodendrocytes after 14 days of cocultivation (Figure 6C,D), indicating their ability to produce myelin. In contrast, transplanted undifferentiated NCSC-derived NSCs located in the white matter showed very low levels of MBP (Figure 6E,F). Quantification of huNu^+^MBP^+^ double positive cells validated the significant increase of human MBP^+^ cells in H_2_O_2_-treated slices after 2 weeks of transplantation with predifferentiated NSCs (10.87% ± 0.73%, +PTXF, Figure 6G) compared to those transplanted with untreated NSCs (5.63% ± 0.64%, −PTXF, Figure 6G, nonparametric Kruskal-Wallis test * *p* < 0.05). However, no significant differences in the ratio of huNu^+^ cells were observable after 2 weeks in H_2_O_2_-treated slices transplanted with predifferentiated NSCs (67.87% ± 7.23%, +PTXF, Figure 6H), compared to untreated NSCs (31.71% ± 16.02%, −PTXF, Figure 6H, nonparametric Kruskal-Wallis test *p* = 0.1266). Taken together, these findings confirmed the ability of PTXF-treated predifferentiated NSCs to give rise to MBP^+^ myelinating oligodendrocytes within the proper neural environment, ex vivo. We therefore demonstrate that PTXF-treated NSCs can attenuate the decrease in MBP observed in the demyelination induced by oxidative stress, further validating their therapeutic potential.

## 4. Discussion

**Figure 7:**
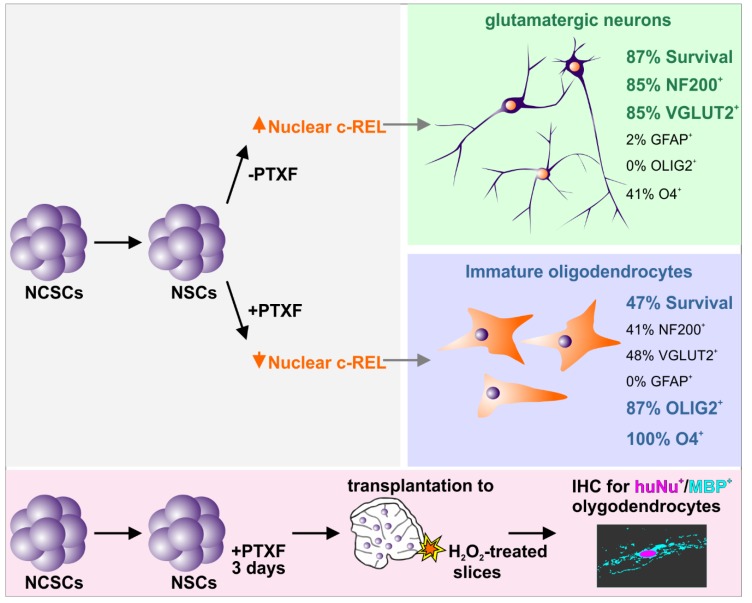
c-Rel inhibition drives hNSCs switch from neuronal to oligodendroglial phenotype. Upper panel: NCSCs are specified to NSCs, and differentiate upon neuronal induction into glutamatergic neurons. Cells undergo a strong increase in nuclear c-REL during early differentiation in the absence of PTXF, that induces a specific gene program expression towards the neuronal fate. Confirmed by an increased in mature neuronal markers NF200 and VGLUT2, and a strong reduction in O4, the absence of GFAP and OLIG2 accompanied by a strong survival, whereas PTXF-treated differentiated NSCs had no increase in nuclear c-REL, leading to a shift in the cell fate into the oligodendrocyte lineage. This was confirmed by a clear increase in oligodendrocyte markers OLIG2 and O4, and a strong reduction in neuronal markers (NF200, VGLUT2), and also accompanied by a strong reduction in cell survival. Bottom panel: Transplantation of PTXF-treated predifferentiated NSCs into a demyelination mouse organotypic cerebellar slice model further demonstrated their capability to differentiate into MBP^+^ oligodendrocytes and produce myelin ex vivo. Cells exhibited a clear oligodendroglial phenotype and differentiated into huNu^+^/MBP^+^ oligodendrocytes after 14 days of cocultivation. PTXF: pentoxifylline, NCSCs: neural crest-derived stem cells, NSCs: neural stem cells.

Our findings demonstrate for the first time that glutamatergic differentiation of human NCSC-derived NSCs is driven by NF-κB-c-REL, while c-REL impairment by pentoxifylline (PTXF) had a strongly significant effect on cell survival, and also led to a direct shift from glutamatergic neurons towards oligodendrocytes. We observed a transient opposing nuclear increase from RELB/p52 to c-REL in an early phase of glutamatergic differentiation, which might be orchestrating fate decisions towards the neuronal fate. While the dynamics observed in the expression pattern from RELB/p52 to c-REL were mediated in an IκBα-dependent manner, RELA and p50 showed no relevant activity during this process. Accordingly, NF-κB-p65 activity was implicated in pluripotency maintenance and decreased upon differentiation of human embryonic stem cells (hESCs) [27,28]. In addition, a similar decrease in NF-κB-p65 allowed differentiation to occur of hESC-derived neural progenitor cells during in vitro neurogenesis [29]. Knockdown of p65 in the initial differentiation phase of hESCs likewise resulted in the upregulation of endodermal and mesodermal markers and mesenchymal specification [30]. In contrast, p65-activation was reported to be increased upon differentiation in mouse embryonic stem cells [31,32]. Despite this well-characterized role of NF-κB-p65 in defining the undifferentiated state in human and mouse embryonic stem cells, p65 seems not to directly mediate cell fate decisions in human stem cells. Accordingly, our present findings define a novel role of NF-κB-c-REL in mediating the differentiation, cell survival and fate shifts of adult human stem cells (Figure 7). In terms of cell survival, we observed a significant increase in apoptosis after 30 days of differentiation in the presence of the c-REL inhibitor PTXF. These results are in accordance with previously described antiapoptotic effects of c-REL in neurons [33] and the regulatory role of NF-κB in cell proliferation [34]. In agreement with our results, PTXF could sensitize Hela and SiHa cells to apoptosis [35], where treatment with 8mM PTXF (2 mg/mL) led to a cell death of about 77%. Here, we used a much lower dose (500 µg/mL), i.e., a c-REL-inhibiting dose for NCSCs.

In addition to the decrease in cell survival, PTXF inhibition of the c-REL peak activity during neuronal differentiation induced a switch from neurogenesis to gliogenesis, into the oligodendrocyte fate (Figure 7). Inhibition of c-REL led to a significant decrease in the amount of NF200^+^ neurons and VGLUT2^+^ neurons compared to control. However, our findings indicate that a minority of these cells coexpress neuronal and oligodendroglial markers. These data suggest that in the used human differentiation system, a common precursor exists that could give rise to oligodendrocytes and also to neurons, as previously shown in the mouse [36]. hNSCs underwent differentiation into a heterogeneous population containing *PDGFRα*^+^/*NG2*^+^-oligendendrocyte precursor cells, immature OLIG2^+^/O4^+^-premyelinating and mature myelinating oligodendrocytes expressing *MBP*, according to the classification of oligodendrocyte-specific markers by Miron and coworkers [5]. A hypothesis for the observed results might be that proneuronal precursors rely on c-REL for differentiation and survival. After inhibition with PTXF, neuronal precursors die, and cells of oligodendrocyte fate are much more represented than in the normal neuronal differentiation. Thus, c-REL might have a negative effect on the oligodendroglial fate. Interestingly, a similar shift from neurogenesis to gliogenesis was observed in the central nervous system by downregulation of the proneural genes required for neuronal differentiation [37]. Hence, the c-REL-mediated neuronal differentiation of hNSCs and the switch towards gliogenesis upon c-REL-inhibition observed here might be dependent on the direct regulation of similar proneural genes. Further investigation in this direction will provide important insights into the possible connection between c-REL and neuronal determination. Despite the robust fate shift towards gliogenesis, hNSCs did not show any signs of GFAP, suggesting the absence of radial glia or no differentiation into astrocytes [38] upon inhibition of c-REL. Accordingly, a strong correlation between the number of oligodendrocytes and neurons was observed in the human brain throughout aging, while the amount of astrocytes remained unchanged [3]. These observations are contrary to those made in the mouse brain, and emphasize the need to address developmental research questions using human model systems [39]. In addition to the differentiation switch established in vitro in the presence of the c-REL inhibitor PTXF, our findings proved that predifferentiated NSCs treated with PTXF and transplanted into demyelinated murine organotypic cerebellar slices, ex vivo, gave rise to huNu^+^/MBP^+^ myelinating oligodendrocytes (Figure 7) within the proper neural environment.

Whereas PTXF treatment had a positive effect on the animal model of multiple sclerosis, Experimental Autoimmune encephalomyelitis [40], it failed as a monotherapy in the translation to multiple sclerosis patients in progression phase [17,41]. One limitation of oral applied PTXF was sterile brain inflammation in rare cases (PTXF ratiopharm product information). In contrast, PTXF combined with interferon beta-1b seem to act synergistically, being beneficial [42]. Besides this known mode of action, our present observations indicate that PTXF may directly modify the cell populations within the central nervous system via c-REL-inhibition in vivo, which is of potential interest for treating not only multiple sclerosis but also schizophrenia, i.e., diseases where the number of oligodendrocytes in the brain is decreased [43]. Emphasizing the role of fate shifts in multiple sclerosis, fibrinogen was recently observed to enter the brain by a damaged blood-brain-barrier and shift oligodendrocyte precursor cell differentiation from oligodendrocytes towards astrocytes, further decreasing remyelination [44,45].

In summary, we demonstrate here an essential role of NF-κB-c-REL in the choice of human NSCs. c-REL inhibition led to a significant increase in apoptosis after 30 days of differentiation, while the remaining cells that did survive switched their fate and differentiated into oligodendrocytes instead. These findings suggest an indispensable role of c-REL during neural differentiation in vivo and a potential function within demyelinating diseases like multiple sclerosis.

## Supplementary Materials

The following are available online at https://www.mdpi.com/2073-4409/9/4/1037/s1, Figure S1: Schematic view on NF-κB family members and their potential effects on cell fate and survival, Figure S2: Immunocytochemical analysis of p65 (RELA), Figure S3: Immunocytochemical analysis of RELB, Figure S4: Immunocytochemical analysis of p52, Figure S5: Immunocytochemical analysis of p50, Figure S6: Immunocytochemical analysis of IκBα, Figure S7. Negative controls for the immunocytochemical analysis of NF-κB subunits, Figure S8: Immunocytochemical analysis of different cell markers after 30 days of glutamatergic neuronal differentiation in the absence or presence of pentoxifylline, Figure S9: Negative controls for the immunocytochemical analysis of cell differentiation markers. Figure S10: Death rate quantified for NCSC-derived NSCs differentiated for 5 days in the absence or presence of PTXF, Table S1: Primers sequences for quantitative polymerase chain reaction.
